# Antiviral Susceptibilities of Distinct Lineages of Influenza C and D Viruses

**DOI:** 10.3390/v15010244

**Published:** 2023-01-15

**Authors:** Emi Takashita, Shin Murakami, Yoko Matsuzaki, Seiichiro Fujisaki, Hiroko Morita, Shiho Nagata, Misa Katayama, Katsumi Mizuta, Hidekazu Nishimura, Shinji Watanabe, Taisuke Horimoto, Hideki Hasegawa

**Affiliations:** 1 Research Center for Influenza and Respiratory Viruses, National Institute of Infectious Diseases, Tokyo 208-0011, Japan; 2 Department of Veterinary Microbiology, Graduate School of Agricultural and Life Sciences, University of Tokyo, Tokyo 113-8657, Japan; 3 Department of Infectious Diseases, Yamagata University Faculty of Medicine, Yamagata 990-9585, Japan; 4 Department of Microbiology, Yamagata Prefectural Institute of Public Health, Yamagata 990-0031, Japan; 5 Virus Research Center, Clinical Research Division, Sendai Medical Center, Sendai 983-8520, Japan

**Keywords:** influenza C virus, influenza D virus, antiviral susceptibility, RNA polymerase inhibitor, baloxavir, favipiravir

## Abstract

The emergence and spread of antiviral-resistant influenza viruses are of great concern. To minimize the public health risk, it is important to monitor antiviral susceptibilities of influenza viruses. Analyses of the antiviral susceptibilities of influenza A and B viruses have been conducted globally; however, those of influenza C and D viruses are limited. Here, we determined the susceptibilities of influenza C viruses representing all six lineages (C/Taylor, C/Yamagata, C/Sao Paulo, C/Aichi, C/Kanagawa, and C/Mississippi) and influenza D viruses representing four lineages (D/OK, D/660, D/Yama2016, and D/Yama2019) to RNA polymerase inhibitors (baloxavir and favipiravir) by using a focus reduction assay. All viruses tested were susceptible to both drugs. We then performed a genetic analysis to check for amino acid substitutions associated with baloxavir and favipiravir resistance and found that none of the viruses tested possessed these substitutions. Use of the focus reduction assay with the genotypic assay has proven valuable for monitoring the antiviral susceptibilities of influenza C and D viruses as well as influenza A and B viruses. Antiviral susceptibility monitoring of all influenza virus types should continue in order to assess the public health risks posed by these viruses.

## 1. Introduction

Influenza viruses are diverse, rapidly changing, enveloped RNA viruses with a widespread global presence. Four types exist: influenza A virus, influenza B virus, influenza C virus, and influenza D virus. Three classes of antivirals have been approved for influenza treatment or prophylaxis in Japan: an M2 inhibitor (amantadine), neuraminidase (NA) inhibitors (oseltamivir, peramivir, zanamivir, and laninamivir), and RNA polymerase inhibitors (baloxavir marboxil and favipiravir). Laninamivir and favipiravir are approved only in Japan. Because favipiravir increases the risk of teratogenicity and embryotoxicity, it received conditional marketing approval with strict regulations for its production and clinical use [1]. The M2 inhibitor blocks the M2 ion channel of influenza A viruses and inhibits virus uncoating [2]. The NA inhibitors bind to the NA enzyme active site of influenza A and B viruses and inhibit virus release from infected cells [2]. The influenza virus RNA-dependent RNA polymerase consists of three subunits: polymerase basic protein 1 (PB1), polymerase basic protein 2 (PB2), and polymerase acidic protein (PA) in influenza A and B viruses or polymerase 3 protein (P3) in influenza C and D viruses. Baloxavir binds to the PA endonuclease domain and inhibits the cap-dependent endonuclease activity of the PA subunit [3]. Favipiravir acts as a chain terminator or a mutagen and inhibits RNA elongation, which is carried out by the PB1 subunit [4].

Antiviral-resistant influenza A viruses have emerged and spread globally. In 2003–2004, the prevalence of M2 inhibitor-resistant A(H3N2) viruses carrying an S31N substitution in the M2 protein increased markedly in China; these viruses spread globally during 2005–2006 [5]. Since the currently circulating influenza A viruses possess the M2 S31N substitution, the World Health Organization (WHO) does not recommend the use of the M2 inhibitors for the treatment or prevention of influenza A virus infections. Influenza A(H1N1) viruses carrying an H275Y substitution in the NA protein, which confers cross-resistance to oseltamivir and peramivir, emerged in Europe during the 2007–2008 influenza season and spread globally within a year. In 2009, influenza A(H1N1)pdm09 viruses, which were susceptible to NA inhibitors, emerged and replaced the NA H275Y mutant A(H1N1) viruses. Although a widespread cluster of baloxavir-resistant viruses has not been detected, human-to-human transmission of influenza A(H3N2) viruses carrying an I38T substitution in the PA protein, which confers resistance to baloxavir, has been reported [6,7,8]. The emergence and spread of antiviral-resistant viruses are of great concern. Because global surveillance of antiviral resistance is essential, the WHO Global Influenza Surveillance and Response System (GISRS) Expert Working Group for Surveillance of Antiviral Susceptibility (WHO-AVWG) has been conducting a global analysis of circulating influenza A and B viruses for NA inhibitor and baloxavir susceptibilities by using a combination of phenotypic methods analyzing antiviral susceptibility and genotypic methods detecting amino acid substitutions associated with antiviral resistance [9].

Influenza C and D viruses contain the surface glycoprotein hemagglutinin-esterase-fusion (HEF), which combines the functions of the hemagglutinin (HA) and NA proteins of influenza A and B viruses. Based on the HEF gene sequences, influenza C and D viruses are classified into six (C/Taylor, C/Yamagata, C/Sao Paulo, C/Aichi, C/Kanagawa, and C/Mississippi) [10] and five (D/OK, D/660, D/Yama2016, D/Yama2019, and D/CA2019) [11] lineages, respectively. The M2 inhibitor and NA inhibitors are not active against influenza C and D viruses. The RNA-dependent RNA polymerase is highly conserved among influenza A, B, C, and D viruses [12]. A few studies have examined the susceptibility of some influenza C and D viruses to baloxavir and favipiravir by using conventional cell-based assays, a plaque reduction assay [13] and a yield reduction assay [14] in Madin-Darby Canine Kidney (MDCK) cells; however, their findings were limited. We have been conducting global and nationwide monitoring of the baloxavir susceptibility of influenza A and B viruses by using a focus reduction assay in MDCK cells and humanized MDCK cells, hCK cells [15], which express high levels of α2, 6-sialoglycans and very low levels of α2, 3-sialoglycans [9,16]. The focus reduction assay is sensitive, robust, and less laborious than conventional cell-based assays and is highly suitable for the surveillance of antiviral susceptibility of influenza viruses [17]. Furthermore, we have successfully applied this assay to severe acute respiratory syndrome coronavirus 2 (SARS-CoV-2) in VeroE6/TMPRSS2 cells and Vero E6-TMPRSS2-T2A-ACE2 cells [18,19,20,21,22]. Here, we determined the susceptibilities of influenza C viruses representing all six lineages and influenza D viruses representing four lineages (D/OK, D/660, D/Yama2016, and D/Yama2019) to baloxavir and favipiravir by using this assay. Since influenza D viruses replicate well in MDCK cells and swine testicular (ST) cells [23], we applied this assay to ST cells. To our knowledge, this is the first report describing the focus reduction assay for influenza C and D viruses and the antiviral susceptibilities of Japanese lineages of influenza D viruses D/Yama2016 and D/Yama2019.

## 2. Materials and Methods

### 2.1. Viruses and Cells

Influenza C viruses were propagated in MDCK cells (NBL-2, ATCC CCL-34) in Dulbecco’s Modified Eagle Medium (DMEM) containing 0.2% bovine serum albumin (BSA) supplemented with 5 μg/mL N-tosyl-L-phenylalanyl chloromethyl ketone (TPCK)-trypsin at 34 °C. Influenza D viruses were propagated in ST cells (ATCC CRL-1746) in DMEM containing 0.2% BSA supplemented with 0.5 μg/mL TPCK-trypsin at 34 °C. The phylogenetic analyses of the influenza C and D viruses used in this study were previously reported [10,24]. D/swine/Oklahoma/1334/2011 and D/bovine/Nebraska/9-5/2012 were kindly provided by Dr. Benjamin Hause (South Dakota State University, USA).

Influenza A(H1N1)pdm09, A(H3N2), and B/Yamagata lineage viruses served as reference wild-type and baloxavir-resistant viruses. Influenza A and B viruses were propagated in MDCK cells containing 0.2% BSA supplemented with 1 μg/mL TPCK-trypsin at 34 °C. Influenza A(H1N1)pdm09 and A(H3N2) viruses possessing the PA I38T substitution were reported in our previous studies [25,26]. RG-B/Yamanashi/166/1998 (wild-type) and RG-B/Yamanashi/166/1998 (PA I38T mutant) were kindly provided by the WHO Collaborating Center for Reference and Research on Influenza, Melbourne.

### 2.2. Antiviral Compounds

The active form of baloxavir marboxil (baloxavir acid) and favipiravir were purchased from Funakoshi Co., Ltd. (Tokyo, Japan) and MedChemExpress (Monmouth Junction, NJ, USA), respectively. Both compounds were dissolved in dimethyl sulfoxide.

### 2.3. Focus Reduction Assay

Baloxavir and favipiravir susceptibilities were determined by using the focus reduction assay as previously described [16] in MDCK cells and ST cells. Since ST cells are more sensitive to TPCK-trypsin than MDCK cells, the assay conditions were modified for ST cells. Briefly, confluent monolayers of cells in 96-well plates were infected with 1000 focus-forming units (FFU) of virus/well. Virus adsorption was carried out for 1 h at 37 °C for efficient binding and then an equal volume of Avicel RC-581 (2.4% in MDCK cells; 1.2% in ST cells, DuPont Nutrition USA, Wilmington, DE, USA) in culture medium containing serial dilutions of baloxavir acid (0.025–2500 nM) or favipiravir (0.01–1000 μM) supplemented with TPCK-trypsin (1.0 μg/mL for influenza A, B, and D viruses or 5.0 μg/mL for influenza C viruses in MDCK cells; 0.5 μg/mL for influenza A, B, C, and D viruses in ST cells) was added to each well in triplicate. The cells were incubated for 18 h at 34 °C and then fixed with formalin. After the formalin was removed, the cells were immunostained with a mouse monoclonal antibody against the nucleoprotein of influenza A (A1 and A3, Merck KGaA, Darmstadt, Germany), B (B2 and B4, Merck KGaA), C (H27) [27], and D (2A5) viruses, followed by a horseradish peroxidase-labeled goat anti-mouse immunoglobulin (SeraCare Life Sciences, Milford, MA, USA). Since the monoclonal antibody 2A5 reacts nonspecifically with ST cells, a mouse monoclonal antibody against the HEF of influenza D virus (1E12) was used in ST cells. The infected cells were stained with TrueBlue Substrate (SeraCare Life Sciences) and then washed with distilled water. After cell drying, the focus numbers were quantified by using an ImmunoSpot S6 Analyzer, ImmunoCapture software and BioSpot software (Cellular Technology, Cleveland, OH, USA). The results are expressed as 50% inhibitory concentration (IC_50_) values, which were calculated by using GraphPad Prism (GraphPad Software, San Diego, CA, USA).

### 2.4. Genetic Analysis

The whole genome sequences of the influenza C and D viruses used in this study have been reported elsewhere [10,24,28]. Isolate IDs in the GISAID EpiFlu Database (https://gisaid.org): C/Ann Arbor/1/50: EPI_ISL_66438; C/Yamagata/15/2004: EPI_ISL_65156; C/Yamagata/32/2014: EPI_ISL_182756; C/Yamagata/4/92: EPI_ISL_230280; C/Yamagata/13/2014: EPI_ISL_182751; and C/Yamagata/3/2000: EPI_ISL_66420. Accession numbers in GenBank: D/swine/Oklahoma/1334/2011: JQ922305 to JQ922311; D/bovine/Nebraska/9-5/2012: KM392468 to KM392474; D/bovine/Yamagata/10710/2016: LC318665 to LC318671; and D/bovine/Yamagata/1/2019: LC494105 to LC494111. Amino acid substitutions associated with resistance to baloxavir and favipiravir were confirmed by using Analyze Sequence Variation (SNP) tool in the Influenza Research Database through the web site at https://www.fludb.org [29].

### 2.5. Statistical Analysis

Statistical analyses were performed using GraphPad Prism and included the unpaired t-test, Mann–Whitney test, and ROUT test. Differences between groups were considered significant for *p* values of <0.05.

## 3. Results

**Figure 1:**
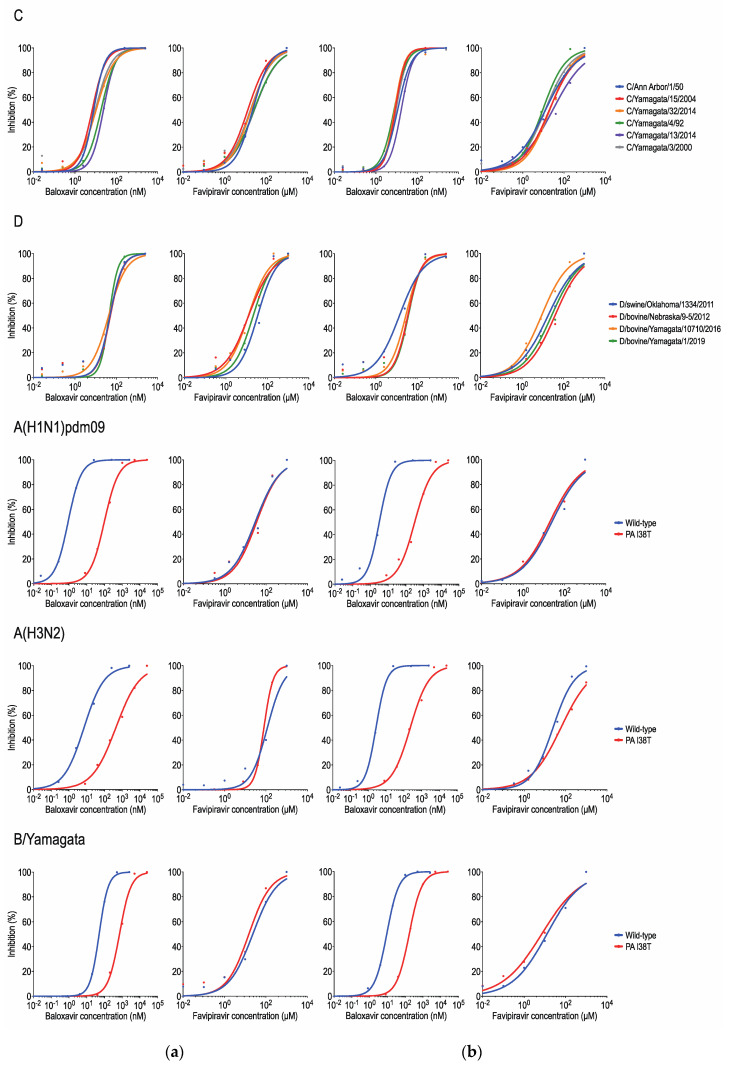
Antiviral activity of RNA polymerase inhibitors against influenza C and D viruses. Baloxavir and favipiravir susceptibilities were determined by using a focus reduction assay in MDCK cells (**a**) and ST cells (**b**). The 50% inhibitory concentration (IC_50_) values for each drug are presented in Table 1. C: influenza C virus; D: influenza D virus; A(H1N1)pdm09: influenza A(H1N1)pdm09 virus; A(H3N2): influenza A(H3N2) virus; B/Yamagata: influenza B/Yamagata lineage virus.

We determined the susceptibilities of all six lineages (C/Taylor, C/Yamagata, C/Sao Paulo, C/Aichi, C/Kanagawa, and C/Mississippi) of influenza C viruses and four lineages (D/OK, D/660, D/Yama2016, and D/Yama2019) of influenza D viruses to baloxavir and favipiravir by using the focus reduction assay in MDCK cells and ST cells (Table 1 and Figure 1).

Criteria to define RNA polymerase inhibitor susceptibility have not yet been established; therefore, provisional criteria based on IC_50_ fold-change thresholds, compared to the median for wild-type viruses of the same type, subtype, and lineage, are used by the WHO-AVWG [9]. The provisional criteria define influenza virus inhibition as normal (<3-fold increase) or reduced (≥3-fold increase). Reference A(H1N1)pdm09, A(H3N2), and B/Yamagata lineage viruses possessing the PA I38T substitution showed 110-, 85-, and 15-fold higher IC_50_ values in MDCK cells and 110-, 91-, and 19-fold higher IC_50_ values in ST cells, respectively, than their corresponding wild-type viruses for baloxavir. However, these I38T mutant viruses had comparable IC_50_ values for favipiravir to their corresponding wild-type viruses in MDCK cells (0.5- to 1.1-fold increase) and ST cells (0.7- to 2.0-fold increase). The wild-type B virus showed higher IC_50_ values for baloxavir than wild-type A(H1N1)pdm09 and A(H3N2) viruses in both cell types, consistent with previous reports using MDCK cells [3,16,30].

Based on the internal gene sequences, six lineages (C/Taylor, C/Yamagata, C/Sao Paulo, C/Aichi, C/Kanagawa, and C/Mississippi) of influenza C viruses and four lineages (D/OK, D/660, D/Yama2016, and D/Yama2019) of influenza D viruses were classified into two groups, respectively; C/Yamagata and C/Mississippi for influenza C viruses [10] and D/OK and D/Yama2016 for influenza D viruses [24]. We found no significant differences in the IC_50_ values for baloxavir among the distinct lineages of influenza C or D viruses. The median IC_50_ values for baloxavir were 10.89 nM in MDCK cells and 10.32 nM in ST cells for influenza C viruses and 48.10 nM in MDCK cells and 31.08 nM in ST cells for influenza D viruses, respectively. The fold-changes in IC_50_ values compared with the median value for tested viruses were 0.6- to 2.3-fold in MDCK cells and 0.8- to 1.7-fold in ST cells for influenza C viruses and 0.9- to 1.1-fold in MDCK cells and 0.4- to 1.2-fold in ST cells for influenza D viruses, respectively. These results suggest that influenza C viruses are more susceptible to baloxavir than influenza D viruses, consistent with a previous study [14]. All Influenza C and D viruses tested had comparable IC_50_ values for favipiravir. The median IC_50_ values for favipiravir were 20.91 μM in MDCK cells and 20.76 μM in ST cells for influenza C viruses and 17.37 μM in MDCK cells and 27.16 μM in ST cells for influenza D viruses, respectively. The fold-changes in IC_50_ values compared with the median value for tested viruses were 0.6- to 1.4-fold in MDCK cells and 0.8- to 2.8-fold in ST cells for influenza C viruses and 0.7- to 2.1-fold in MDCK cells and 0.4- to 1.7-fold in ST cells for influenza D viruses, respectively. The IC_50_ values of the reference B viruses for favipiravir were similar to those of influenza C viruses, as previously reported [13], suggesting that favipiravir susceptibilities of influenza B viruses are approximately comparable to those of influenza C and D viruses.

The I38T substitution in the PA protein that is associated with baloxavir resistance emerged after baloxavir treatment in the Phase II and III clinical trials of baloxavir [3,31,32,33]. No mutant viruses with reduced susceptibility to favipiravir emerged after favipiravir treatment in clinical trials of favipiravir; however, in vitro studies have shown that a K229R substitution in the PB1 protein confers about a 30-fold reduction in favipiravir susceptibility of influenza A viruses [34]. We therefore checked for amino acid substitutions associated with resistance to baloxavir and favipiravir in the influenza C and D viruses used in this study (Figure S1). The P3 and PB1 gene sequences of the viruses were screened for amino acid substitutions at position 38 in P3 and position 231 in PB1, which corresponds to position 229 in influenza A viruses. None of the influenza C and D viruses tested possessed the P3 38T or PB1 231R substitutions. However, the amino acid at position 38 of influenza D virus P3 was found to be valine, which was recently reported to confer a 3.0- to 3.7-fold increase in IC_50_ values to baloxavir [9].

## 4. Discussion

Influenza C and D viruses are genetically more closely related to each other than to influenza A or B viruses, suggesting a common ancestor for C and D viruses [35]. Influenza C virus was first isolated from humans in 1947 [36] and is widely distributed throughout the world. It infects humans, dogs, swine, camels, horses, and bovines, although humans are the primary host [37]. Influenza C virus causes relatively mild respiratory illness; however, it can cause bronchitis and pneumonia, particularly in children younger than 2 years or patients with underlying illnesses [38,39,40,41,42]. Influenza D virus was first detected in swine in 2011 [23]. Since then, the virus has been detected in bovines in several countries including USA, Mexico, France, Japan, Italy, China, and Ireland [43,44,45,46,47,48]. Global serological studies have shown that influenza D virus has a broad host range, including humans, swine, sheep, goats, camels, horses, and bovines, although the primary host of influenza D virus is bovines [37].

Influenza D viruses, as well as influenza C viruses, use 9-O-acetylated sialic acids (Neu5,9Ac2) as the host cell receptor [49] and replicate efficiently in a human well-differentiated respiratory epithelium model (i.e., human airway epithelial cells: hAECs) [50]. Influenza D virus genomes have been detected in one (2.3%) of 44 or one (4.2%) of 24 bioaerosol samples from a hospital emergency room [51] and an airport [52], respectively in North Carolina, USA and in one (1.3%) of 78 nasal washes from pig farm workers in Sarawak, Malaysia [53]. Moreover, a high seroprevalence (94–97%) of antibodies to influenza D virus was detected among cattle workers in Florida, USA [54]. These observations indicate that influenza D virus has the potential to cause zoonotic infections. Although confirmed cases of human infections with influenza D virus have not been reported, the public health risks of this virus should be assessed.

At present, there are no antiviral drugs approved for influenza C or D virus infections, although antivirals are widely used for the treatment or prophylaxis of influenza A and B virus infections. Since the RNA-dependent RNA polymerase is highly conserved among influenza A, B, C, and D viruses, RNA polymerase inhibitors, such as baloxavir and favipiravir, may be active against influenza C and D viruses. Baloxavir was approved for the treatment of influenza A and B virus infections in Japan and the USA in 2018 and has since been approved in several other countries; favipiravir was approved for influenza pandemic preparedness in Japan in 2014 and stockpiled for use against novel influenza virus infections for which first-line antivirals are ineffective [1]. Influenza A viruses are known to be more susceptible to baloxavir than influenza B viruses [3,16,30]. In this study, we found that all influenza C and D viruses tested had IC_50_ values comparable to or lower than the reference wild-type A and B viruses for baloxavir. These results suggest that baloxavir could be a potential drug for the treatment of influenza C and D virus infections. Clinical data on the efficacy of this drug in patients infected with influenza C and D viruses are needed in the future.

Baloxavir acid, an active form of baloxavir marboxil, forms van der Waals interactions with A20, Y24, K34, A37, and I38 in influenza A virus or with T20, F24, M34, N37, and I38 in influenza B virus in the PA endonuclease domain [3]. Although the structure of the P3 endonuclease domain of influenza C and D viruses co-crystalized with baloxavir acid has not been analyzed, amino acid differences at these positions may be related to the lower baloxavir susceptibilities: I20, A24, K34, Q37, and I38 in influenza C virus or I20, V24, T34, S37, and V38 in influenza D virus. In fact, the I38V substitution was recently reported to be associated with reduced susceptibility to baloxavir [9]. In vitro studies to identify amino acid substitutions associated with RNA inhibitor resistance in influenza C and D viruses may be useful for understanding the differences in IC_50_ values among influenza A, B, C, and D viruses.

The WHO-AVWG has been conducting global analysis of antiviral susceptibilities of circulating influenza A and B viruses since 2012–2013 period [9]. To determine the baloxavir and favipiravir susceptibilities of influenza C and D viruses, we applied the focus reduction assay we use for our global and nationwide monitoring of the influenza A and B viruses to influenza C and D viruses. Using a combination of this focus reduction assay and a genotypic assay to detect amino acid substitutions associated with antiviral resistance has proven valuable for monitoring the antiviral susceptibilities of influenza C and D viruses. Recently, a tentative new lineage of influenza D viruses (the D/Bursa2013 lineage) was detected in cattle in Turkey [55]. Monitoring of the antiviral susceptibilities of influenza viruses should continue in order to assess the public health risks posed by these viruses.

## Figures and Tables

**Table 1:** Susceptibilities of influenza C and D viruses to RNA polymerase inhibitors.

Type	Subtype/Lineage	Virus	Mean IC_50_ ± SD *			
			MDCK Cells		ST Cells	
			Baloxavir (nM)	Favipiravir (μM)	Baloxavir (nM)	Favipiravir (μM)
C	C/Taylor	C/Ann Arbor/1/50	7.73 ± 2.67	22.37 ± 3.58	11.29 ± 0.58	20.07 ± 10.00
	C/Yamagata	C/Yamagata/15/2004	6.20 ± 0.92	13.20 ± 1.15	8.87 ± 1.90	21.45 ± 3.46
	C/Sao Paulo	C/Yamagata/32/2014	8.49 ± 3.75	19.44 ± 3.45	11.45 ± 1.26	16.16 ± 5.18
	C/Aichi	C/Yamagata/4/92	19.74 ± 3.39	24.72 ± 1.38	8.19 ± 1.87	16.86 ± 5.63
	C/Kanagawa	C/Yamagata/13/2014	13.28 ± 3.89	17.54 ± 2.41	9.35 ± 2.41	58.34 ± 14.33
	C/Mississippi	C/Yamagata/3/2000	24.83 ± 9.48	28.62 ± 4.85	17.48 ± 6.00	22.22 ± 6.47
D	D/OK	D/swine/Oklahoma/1334/2011	49.83 ± 5.02	35.83 ± 12.83	13.91 ± 1.48	20.81 ± 1.27
	D/660	D/bovine/Nebraska/9-5/2012	51.02 ± 12.11	13.68 ± 3.55	34.76 ± 1.94	46.86 ± 8.63
	D/Yama2016	D/bovine/Yamagata/10710/2016	44.51 ± 9.12	12.48 ± 2.71	27.40 ± 3.22	10.21 ± 1.47
	D/Yama2019	D/bovine/Yamagata/1/2019	46.36 ± 6.47	21.05 ± 1.49	36.75 ± 5.86	33.51 ± 4.52
A	A(H1N1)pdm09	A/Kanagawa/ZC1931/2019 (wild type)	0.88 ± 0.12	31.07 ± 7.86	3.09 ± 0.39	57.96 ± 8.90
		A/Kanagawa/IC1890/2019 (PA I38T) †	98.19 ± 10.33	35.07 ± 6.34	329.50 ± 48.38	40.65 ± 15.85
	A(H3N2)	A/Yokohama/136/2018 (wild type)	6.70 ± 0.65	136.20 ± 41.54	2.65 ± 0.20	30.38 ± 7.83
		A/Yokohama/133/2018 (PA I38T) †	568.03 ± 251.22	73.87 ± 9.86	240.80 ± 71.37	59.65 ± 7.27
B	B/Yamagata	RG-B/Yamanashi/166/1998 (wild type)	46.79 ± 2.93	23.29 ± 1.10	9.09 ± 2.88	19.44 ± 2.89
		RG-B/Yamanashi/166/1998 (PA I38T) †	680.97 ± 21.18	15.56 ± 5.54	169.30 ± 32.24	34.00 ± 5.56

IC_50_: 50% inhibitory concentration; PA: polymerase acidic subunit; RG: reverse genetics. * The mean IC_50_ values of triplicate reactions were determined by using a focus reduction assay in MDCK cells or ST cells. † The I38T amino acid substitution in PA is associated with baloxavir resistance.

## Data Availability

Not applicable.

## Supplementary Materials

The following supporting information can be downloaded at: https://www.mdpi.com/article/10.3390/v15010244/s1, Figure S1: Amino acid alignment of selected regions of the P3 (a) and PB1 (b) proteins of the influenza C and D viruses used in this study.
