# Supplementary material for: Antiviral Susceptibilities of Distinct Lineages of Influenza C and D Viruses
Source: Viruses. 2023 Jan 15;15(1):244. doi: 10.3390/v15010244 (PMC9861540; doi:10.3390/v15010244)
Supplement: Supplementary file 1 [file viruses-15-00244-s001.zip › viruses-2143142-supplementary.pdf]

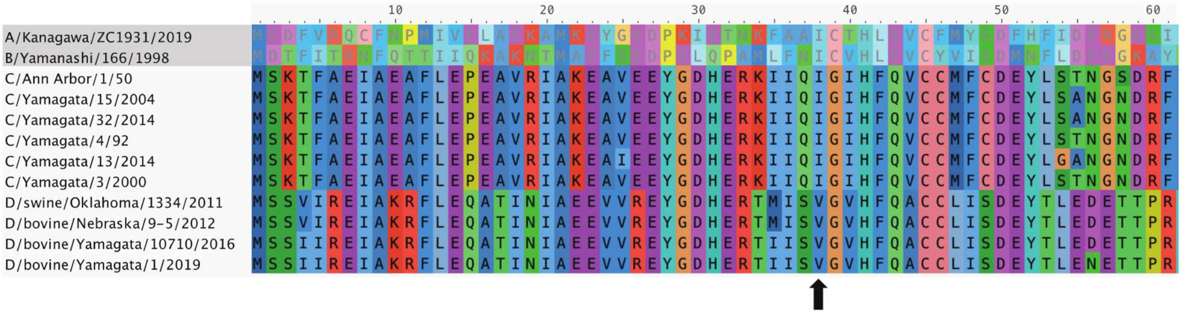


(a)

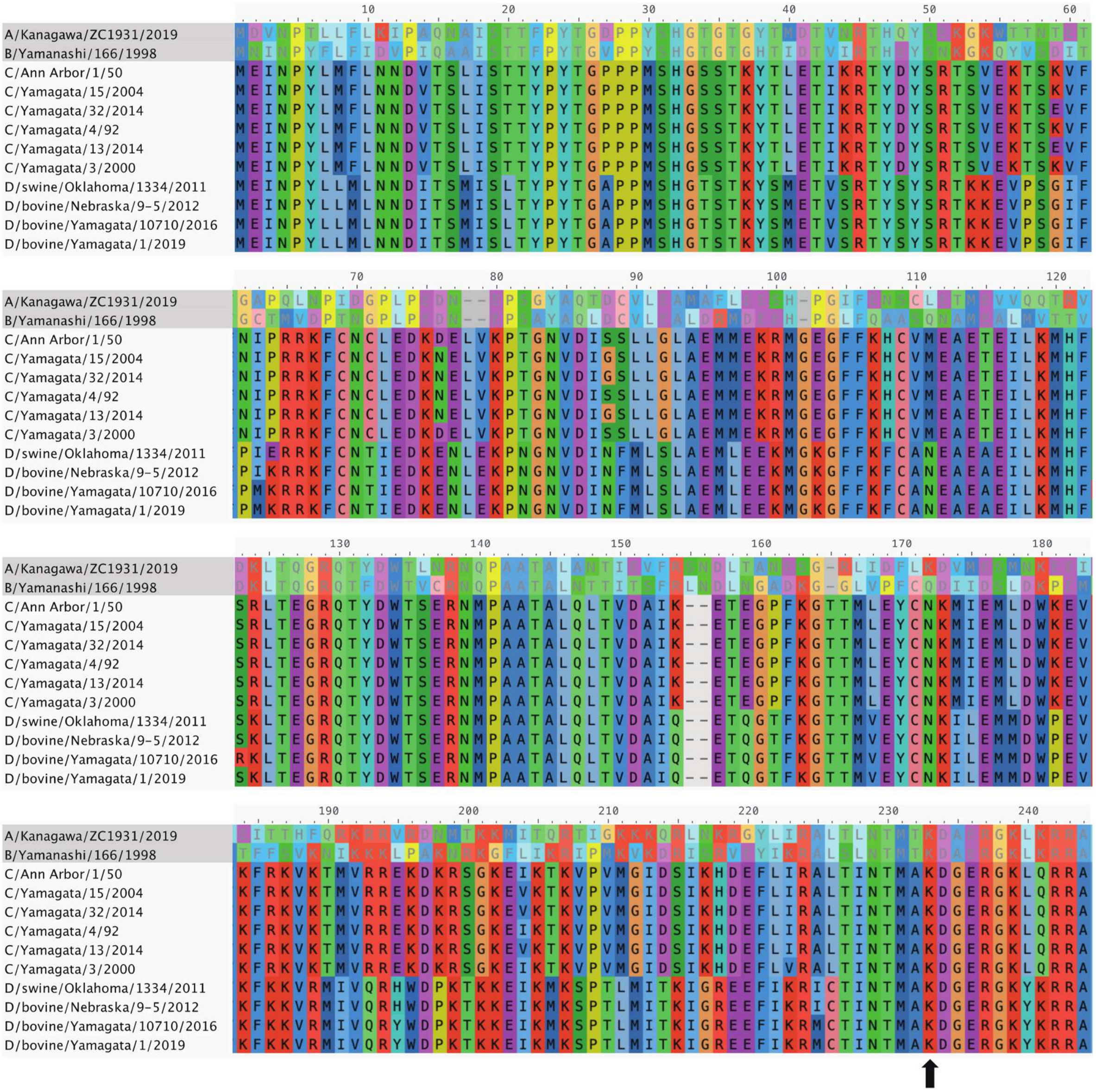


(b)

**Figure S1.** Amino acid alignment of selected regions of the P3 (a) and PB1 (b) proteins of the influenza C and D viruses used in this study. A/Kanagawa/ZC1931/2019(H1N1)pdm09 and B/Yamanashi/166/1998 were used as reference strains. Isolate IDs of A/Kanagawa/ZC1931/2019(H1N1)pdm09 and B/Yamanashi/166/1998 in the GISAID EpiFlu Database (https://gisaid.org) are EPI_ISL_403549 and EPI_ISL_20976, respectively. Arrows indicate positions 38 and 231, respectively.
